# Protocol for LASER: A Randomized Evaluation and an Associated Registry of Early Anticoagulation With Edoxaban After Ischemic Stroke in Patients With Atrial Fibrillation

**DOI:** 10.3389/fneur.2021.645822

**Published:** 2021-03-31

**Authors:** Anas Alrohimi, Glen Jickling, Thomas Jeerakathil, Ashfaq Shuaib, Khurshid Khan, Mahesh Kate, Michael D. Hill, Brian Buck, Ken Butcher

**Affiliations:** ^1^Department of Medicine, University of Alberta, Edmonton, AB, Canada; ^2^Department of Medicine, King Saud University, Riyadh, Saudi Arabia; ^3^Clinical Neurosciences, University of Calgary, Calgary, AB, Canada; ^4^Prince of Wales Clinical School, University of New South Wales, Sydney, NSW, Australia

**Keywords:** atrial fibrillation, ischemic stroke, hemorrhagic transformation, edoxaban, randomized clinical trial

## Abstract

**Background:** The optimal timing of anticoagulation after stroke in patients with atrial fibrillation (AF) is unknown.

**Aim and Hypothesis:** Our primary aim is to demonstrate the safety of edoxaban initiation within 5 days of AF related stroke. Our secondary aim is to determine predictors of hemorrhagic transformation (HT) after AF related stroke. We hypothesize that the rate of radiological HT will not be increased in patients starting edoxaban within 5 days of AF related stroke, relative to those in whom initiation is delayed. We hypothesize that the risk of HT in patients treated with edoxaban can be predicted using RNA expressed in leukocytes at time of stroke.

**Methods and Design:** LASER (Lixiana Acute Stroke Evaluation Registry) is a randomized controlled trial with an associated registry (clinicaltrials.gov NCT03494530). One hundred and fifty patients with ischemic stroke and AF will undergo baseline Computed Tomography (CT) scan and will be randomized 2:1 within 5 days of symptom onset to early (≤5 days, *n* = 100) or delayed (6–14 days, *n* = 50) edoxaban initiation. Participants will undergo clinical assessment and repeat CT at 7 days and clinical assessment at 90 days.

**Study Outcomes:** The primary outcome is the rate of incident radiological HT. Secondary outcomes include symptomatic HT, recurrent ischemic stroke, recurrent sub-clinical infarcts on follow up CT, systemic hemorrhagic complication rate, National Institute of Health Stroke Scale and modified Rankin Scale at day 7 and 90, mortality within 90 days, quality of life assessments at day 90, and predictors of HT, including RNA expression by 6 pre-selected candidate genes.

**Discussion:** Event rates for both HT and recurrent ischemic events, in patients treated with early vs. delayed edoxaban initiation are unknown. The primary study endpoint of LASER is an objective performance criterion relevant to clinical decision making in patients with AF related stroke. This study will provide data required for a definitive safety/efficacy study sample size power calculation.

## Introduction

The optimal timing of anticoagulation after ischemic stroke is an area of clinical equipoise. It is clearly established that patients with atrial fibrillation (AF) who develop ischemic stroke are at high risk for recurrence and require long-term anticoagulation. Previous studies have reported that starting older anticoagulants within 48 h after acute ischemic stroke is associated with a reduction in the rate of recurrent ischemic events, but that is offset by an increased risk of hemorrhagic transformation (HT) (1–3). More recently, four direct oral anticoagulation (DOACs) have shown a lower risk of intracranial hemorrhagic complications compared to older anticoagulants, and they are now the standard of care for long-term stroke prevention in non-valvular AF (4–7). In the pivotal phase III DOAC trials, patients were not eligible for randomization as early as 7 days and up to 30 days after ischemic stroke. Current stroke guidelines are inconsistent regarding the timing of DOAC initiation and recommendations are based on expert opinion only (8–10).

Edoxaban is one of the DOACs approved for the prevention of ischemic stroke in patients with AF (7). Patients within 30 days of ischemic stroke were excluded from the Effective Anticoagulation with Factor Xa Next Generation in Atrial Fibrillation - Thrombolysis in Myocardial Infarction 48 (ENGAGE AF-TIMI 48) trial. As with other DOACs, there are no randomized data related to the use of edoxaban early after ischemic stroke. The optimal timing of DOAC initiation is unknown and a clinical problem commonly encountered by stroke physicians. Even less is understood about the timing of anticoagulation after HT, asymptomatic or otherwise, has occurred.

Symptomatic HT remains difficult to predict. Although clinical severity and infarct volume appear to increase HT risk (11, 12), the association is highly inconsistent. Novel biomarkers would be useful in determining DOAC timing. Preliminary data suggest the risk of HT in patients with stroke can be stratified by RNA expressed in circulating leukocytes within 3 h of stroke onset. A panel of 6 genes associated with subsequent HT has been identified (13, 14).

The primary aim of the Lixiana Acute Stroke Evaluation Registry (LASER) is to demonstrate the safety of edoxaban initiation within 5 days of AF related stroke. Secondary aims include identification of clinical, imaging and RNA transcript predictors of HT. We hypothesize that edoxaban initiation within 5 days of ischemic stroke will not be associated with increased HT rates, relative to patients in whom anticoagulation is delayed. Serial imaging using Computed Tomography (CT) will be utilized to determine the rate of radiological HT after edoxaban initiation. Incident radiological HT rates will be assessed as objective performance criteria for the safety of early vs. delayed edoxaban initiation (15–17). We also hypothesize that RNA expressed in leukocytes at time of stroke can stratify risk of HT in patients treated with edoxaban. We will assess the rate of recurrent ischemic stroke, but recognize any differences between groups will be hypothesis generating only due to the small trial sample size.

## Methods

### Study Design

**Figure 1:**
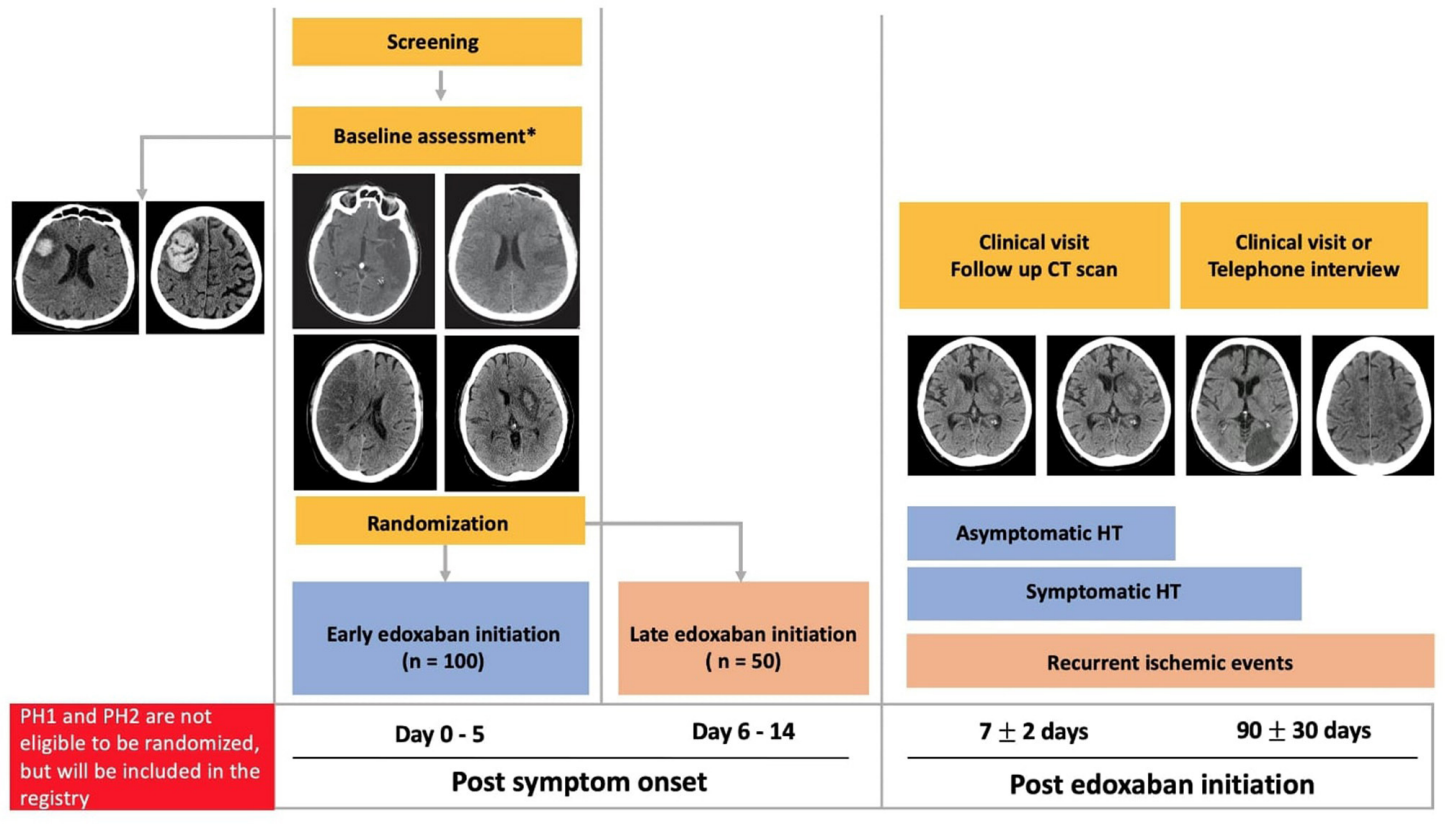
Randomized trial and registry schema. PH, parenchymal hematoma; CT, computed tomography; HT, hemorrhagic transformation. *All stroke severities, infarction sizes and HT (Hemorrhagic infarction type 1 and Hemorrhagic infarction type 2) are eligible for randomization.

LASER is a randomized controlled, parallel-group, two-arm, assessor-blinded trial with an associated registry (clinicaltrials.gov NCT03494530). Patients with previously known or newly diagnosed AF-related ischemic stroke will be randomized 2:1 to early (≤5 days) or delayed (6–14 days) edoxaban initiation (Figure 1). Ischemic stroke will be defined as evidence of acute focal cerebral infarction confirmed on CT/MRI and/or focal hypoperfusion/vessel occlusion on multimodal CT, or by sudden focal and objective neurological deficits (i.e., NIHSS ≥ 1) of presumed ischemic origin persisting > 24 h. Informed consent will be obtained from the patient or substitute decision maker in all cases prior to enrolment. The research protocol has been approved by our local Human Research Ethics Board.

### Patient Population

One hundred fifty patients from a Comprehensive Canadian Stroke Center will be enrolled. Eligible patients will be randomized within 5 days of symptom onset after baseline CT. Inclusion and exclusion criteria are shown in Table 1.

**Table 1:** Study inclusion and exclusion criteria.

**Inclusion criteria**
Male or female patients
≥18 years of age
Ischemic stroke, diagnose and enroll ≤ 5 days from symptom onset^*^
AF (paroxysmal or persistent), confirmed with ECG/Holter monitor, or by history (clinical documentation of previous AF must be provided)
Informed consent
**Exclusion criteria**
Acute or chronic renal failure, defined as eCrCl <30 ml/min (Cockcroft Gault formula)
Known hypersensitivity to edoxaban
Any significant ongoing systemic bleeding risk, or recent major surgery
Recent past history or clinical presentation of ICH, SAH, AVM, aneurysm, or cerebral neoplasm
Hereditary or acquired haemorrhagic diathesis
Stroke mimics
HT with a grade of PH1 or PH2 on baseline or screening CT†
Any condition that, in the judgment of the investigator(s), could impose hazards to the patient if study therapy is initiated

* *Ischemic stroke is defined as evidence of acute focal cerebral infarction confirmed on CT/MRI and/or focal hypoperfusion/vessel occlusion on multimodal imaging, or by sudden focal and objective neurological deficits (i.e., NIHSS ≥ 1) of presumed ischemic origin persisting > 24 h*.

† *Eligible for registry*.

*AF, atrial fibrillation; ECG, electrocardiography; eCrCl, estimated creatinine clearance; ICH, intracerebral hemorrhage; SAH, subarachnoid hemorrhage; AVM, arteriovenous malformation; HT, hemorrhagic transformation; PH, parenchymal hemorrhage; CT, computed tomography*.

Patients with spontaneous parenchymal hemorrhage (PH) [European Cooperative Acute Stroke Study (ECASS)] grade PH1 or PH2 on the baseline CT will not be eligible for randomization (18). These patients will be included in the registry portion of LASER and follow-up will be identical to that in the trial. The timing of edoxaban initiation in these patients will be at the discretion of the treating physician.

### Randomization

Eligible patients will be randomized 2:1 following open label simple randomization procedure to early (≤ 5 days) or delayed (6–14 days) edoxaban initiation via a centralized web-based randomization process, Research Electronic Data Capture (REDCap, Vanderbilt university) (19, 20). After randomization to early or delayed arms, the decision to time the edoxaban initiation within the specific arm will be at the treating physician's discretion.

The rationale for the specific timing of treatment, within the randomization window, will also be recorded by surveying the treating physician in each case.

### Treatment

Randomized patients will be treated with edoxaban 60 mg once daily. The edoxaban dose will be reduced to 30 mg once daily if any of the following characteristics are present at the time of randomization or during the study: estimated creatinine clearance (CrCl) of 30–50 ml per min using Cockcroft-Gault Equation or body weight ≤ 60 kg. Prior to edoxaban initiation, all patients will be treated as per the clinical standard of care using antiplatelet(s) and anticoagulation for deep venous thrombosis (DVT) prophylaxis. Any antithrombotic therapy prior to randomization will be recorded.

### Clinical Assessment

All randomized patients will be followed for 90 days after edoxaban initiation. A National Institute of Health Stroke Scale (NIHSS) score will be assessed at baseline, 7 and 90 days after edoxaban initiation. Functional outcome will be assessed with a modified Rankin Scale (mRS) score at baseline, 7 and 90 days after edoxaban initiation. Montreal Cognitive Assessment (MoCA) will be performed at baseline, and day 90 after edoxaban initiation. Functional outcome at 90 days will be dichotomized as favorable (mRS score 0–2) and unfavorable (mRS score 3–6). Quality of life will be assessed with the EuroQol-5 Dimension (EQ-5D) and Visual Analog Scale (VAS) at day 90.

### Standardized Image Acquisition and Central Analysis

In addition to diagnostic imaging, all patients will have a baseline CT prior to randomization and follow up CT 7 ± 2 days after edoxaban initiation. Therefore, all patients will have a minimum of two scans (diagnostic and pre-randomization). In the event of clinical deterioration, CT scans will be repeated. In patients treated with thrombolysis and/or endovascular thrombectomy, a 24-h post-treatment CT will be used as the baseline study prior to randomization.

### Imaging Protocol

The original LASER protocol included MRI acquisition in all patients. Following diagnostic CT, all patients were to undergo MRI including diffusion-weighted imaging (DWI to assess the acute infarct volume), Fluid Attenuated Inverse Recovery (FLAIR to assess chronic infarct and white matter ischemic change volumes) and susceptibility weighted imaging (SWI to assess for acute HT and chronic cerebral microbleeds) prior to randomization.

Shortly after trial initiation, however, COVID-19 restrictions limited access to research MRI protocols. The protocol was therefore amended and both baseline and day 7 imaging will be performed using non-contast, axial CT.

All CT scans will be assessed by two independent raters, blinded to treatment group, for the presence, number and total volume of regions with infarction. Infarct volumes will be measured using planimetric techniques (Analyze 11.0, Biomedical Imaging Resource, Mayo Clinic) (21). Any HT, as well as other intracranial hemorrhage, seen at baseline and day 7 will be graded using the Heidelberg Bleeding Classification (HBC) (22). Incident HT seen on follow-up CT scan is defined as new or progressive HT. Progressive HT will be defined as any increase in the severity grade between the baseline and follow-up scan. Two raters will rate the HT using the Heidelberg criteria.

### RNA Analysis

A blood sample will be drawn into a PAXgene tube for RNA analysis at the time of the baseline CT. RNA will be isolated and measured by RNA sequencing and reverse transcription polymerase chain reaction (RT-PCR). Genes different between patients who develop HT compared to those without HT will be identified by analysis of variance adjusted for covariates as previously described (13, 14). A prediction model will be developed using the identified genes. The ability of the developed gene model to predict HT will be compared to other factors associated with HT including age, stroke severity, infarct volume.

## Study Outcomes

The primary study endpoint is the rate of incident (new or progressive) radiological HT. The secondary endpoint is the rate of symptomatic HT, defined as intracerebral hemorrhage within and beyond infarcted brain tissue with PH > 1/3 the volume of the ischemic infarct (Class 2 in HBC) associated with clinical deterioration (worsening of NIHSS score by ≥4 points) within 30 days of treatment initiation (18, 22). Other secondary endpoints are recurrent ischemic stroke within 90 days of randomization, recurrent sub-clinical infarcts on follow up CT at 7 ± 2 days post edoxaban initiation, systemic hemorrhagic complication rate within 90 days of randomization, NIHSS at day 7 and 90, mRS score at day 7 and 90, favorable mRS at day 90, and mortality within 90 days, quality of life at 90 days assessed by EQ-5D and VAS, and ability of leukocyte RNA to predict HT.

## Sample Size Estimates

Based on previous open label studies of DOAC use in acute stroke, the rate of symptomatic HT is likely to be nil, which is why we have made this a secondary endpoint. We have previously demonstrated the rate of asymptomatic HT in serial imaging studies to be as lower as 3% in CT-based studies (23, 24) and up to 13% in MRI based studies (25). There are no data related to the difference in asymptomatic HT rates in patients randomized to early vs. late DOAC initiation. Our aim is to determine the event rates in these two groups with reasonable precision.

A sample size of 150 patients, randomized 2:1 (≤5 days:6–14 days) will allow detection of an asymptomatic hemorrhage rate of 3% (95% CI 0.6–8.5%) in the early treatment arm (*n* = 100) and 4% (95% CI 0.5–13.7%) in the late treatment arm (*n* = 50). Thus, we will have confidence interval width of a maximum of 13.7%, allowing enough precision to demonstrate the safety of early treatment. The rationale for adopting a 2:1 randomization approach is to increase the precision around the estimate of safety in the early treatment arm, without substantively losing power.

## Statistical Analysis

The primary analysis will be intention-to-treat, irrespective of edoxaban compliance. The primary endpoint of incident HT rates (and 95% CIs) in the early vs. delayed groups will be calculated. Crude rates will be adjusted only if there are baseline imbalances in stroke severity (NIHSS), infarct volume or HT at baseline. Unadjusted differences in proportions between the two groups will be tested using a Fisher's exact test. Univariate linear and logistic regression analyses will be used to assess potential relationships between CT and clinical factors including age, clinical severity (NIHSS score), history of diabetes/glucose level, hypertension/blood pressure, bridging anti-thrombotic therapy, thrombolysis or endovascular thrombectomy and HT risk.

Confirmed genes will be used to create a linear discriminant analysis prediction model to stratify risk of HT. The model will be assessed by 10-fold leave one out cross-validation. Using logistic regression, the gene prediction of HT will be compared to clinical factors used to stratify risk of HT in stroke including age, stroke severity, blood pressure, glucose, thrombolysis as we have previously described (13, 14).

## Discussion

This randomized controlled trial (RCT) is the first step in advancing the knowledge of the safety of early vs. delayed anticoagulation after ischemic stroke in patients with AF. In our pilot safety studies with rivaroxaban, dabigatran, and apixaban we observed low rates of symptomatic HT when these DOACs were initiated within 14 days of symptom onset (23–25). While encouraging with respect to the safety of early DOAC initiation, these studies are far from definitive. Several trial protocols have been published and/or registered for the safety and efficacy of early vs. delayed anticoagulation after ischemic stroke in patients with AF (26–28). Patients in these trials are randomized to DOAC initiation as early as 24 h and up to 5 days after onset, or delayed initiation 6–14 days. The primary endpoint in these trials is the composite of recurrent ischemic stroke and symptomatic HT. Patient allocation to the treatment arms in most of these trials is determined by stroke severity and/or size. Ongoing trials include TIMING (Timing of Oral Anticoagulant Therapy in Acute Ischemic Stroke With Atrial Fibrillation: a Prospective Multicenter Registry-based Non-inferiority Randomized Controlled Clinical Trial, NCT02961348) (27), ELAN (Early vs. Late Initiation of Direct Oral Anticoagulants in Post-ischaemic Stroke Patients With Atrial fibrillation, NCT03148457), START (Optimal Delay Time to Initiate Anticoagulation After Ischemic Stroke in Atrial Fibrillation, NCT03031928) trials, OPTIMAS (OPtimal TIming of Anticoagulation After Acute Ischemic Stroke, NCT03759938) and AREST (Apixaban for Early Prevention of Recurrent Embolic Stroke and Hemorrhagic Transformation, NTC02283294).

Edoxaban is the newest drug in the DOAC class. Although equipoise exists with respect to timing of initiation of all the DOACs, a design utilizing all four drugs introduces additional confounding factors. A novel aspect of this RCT is to identify RNA transcript as well as clinical and imaging predictors of HT after AF related stroke. If a biomarker of HT risk can be identified, it may be useful in guiding anticoagulation timing after acute cardioembolic stroke. A standardized documentation of HT rates on follow-up CT as objective assessment criteria will be employed in this study. This method is missing from most studies of early anticoagulation.

### Limitations

The amended protocol limits follow-up imaging to CT, making it highly likely that we will under-estimate the true rate of recurrent sub-clinical infarcts. This trial is not powered to detect differences in clinical outcomes, including symptomatic HT and recurrent infarction. Dichotomization at 5 days leaves a considerable discretionary range within which clinicians can initiate edoxaban. In cases where the period of equipoise is narrower, i.e., day 0 vs. day 2–3, this may be relevant. Stratified randomization can address this to an extent, but as we are assessing radiological HT event rates, a more pragmatic approach was chosen for this trial.

### Trial Status

Enrolment of patients started in November 2018 and study completion is estimated in December 2023. Protocol version 4.0 was approved on 14 January 2021.

## Conclusion

Objective evaluation criteria such as systematically acquired imaging assessment of HT is required to understand the true effect of DOAC timing. These data are critical to developing future efficacy trials. Accurate and objective predictors of HT, including novel biomarkers, will also help refine future trial inclusion/exclusion criteria.

## Ethics Statement

This protocol was approved by University of Alberta Human Research Ethics Committee. The patients/participants or substitute decision maker will provide written informed consent to participate in this study.

## Author Contributions

AA drafted the manuscript. KB designed the protocol, obtained grant funding and is overseeing trial conduct. GJ is overseeing all mRNA analyses and wrote these sections of the protocol and the manuscript. BB is the site lead at the University of Alberta, contributed to protocol development and made critical revisions of the manuscript. MH and TJ wrote the statistical analysis sections of the manuscript. AS, KK, and MK made critical revisions of the manuscript. All authors contributed to the article and approved the submitted version.

## Conflict of Interest

The authors declare that the research was conducted in the absence of any commercial or financial relationships that could be construed as a potential conflict of interest.
